# Perceived performance of activities of daily living by stroke patients: key in decision to call EMS and outcomes

**DOI:** 10.3389/fneur.2023.1241391

**Published:** 2023-09-22

**Authors:** Luca Tarantini, Fatma Merzou, Maxine Luley, Aline Rollmann, Michael Peter Schwindling, Martin Lesmeister, Marietheres Gläss, Jennifer Wollenburg, Lenka Schwindling, Klaus Fassbender

**Affiliations:** ^1^Experimental Neuropsychology Unit, Department of Psychology, Saarland University, Saarbrücken, Germany; ^2^Department of Psychiatry and Psychotherapy, Saarland University Medical Center, Homburg, Germany; ^3^Department of Neurology, Saarland University Medical Center, Homburg, Germany

**Keywords:** stroke, public education, help-seeking, prehospital, emergency medical service (EMS), psychological

## Abstract

**Background:**

Until recently, public education campaigns aimed at improving help-seeking behavior by acute stroke patients have achieved only limited or even no effects. Better understanding of psychological factors determining help-seeking behavior may be relevant in the design of more effective future campaigns.

**Methods:**

In this prospective, cross-sectional study, we interviewed 669 acute stroke patients within 72 h after hospital admission. The primary endpoint was the effect of psychological factors on the decision to call emergency medical services (EMS). Secondary endpoints were the effects of such factors on treatment rates and clinical improvement (difference between modified Rankin scale (MRS) scores at admission and at discharge).

**Results:**

Only 48.7% of the study population called the EMS. Multivariate logistic and linear regression analyses revealed that perception of unimpaired performance of activities of daily living (ADL) was the only psychological factor that predicted EMS use and outcomes. Thus, patients who perceived only minor impairment in performing ADL were less likely to use EMS (odds ratio, 0.54 [95% confidence interval, 0.38–0.76]; *p* = 0.001), had lower treatment rates, and had less improvement in MRS scores (*b* = 0.40, *p* = 0.004). Additional serial mediation analyses involving ischemic stroke patients showed that perception of low impairment in ADL decreased the likelihood of EMS notification, thereby increasing prehospital delays, leading to reduced thrombolysis rates and, finally, to reduced clinical improvement.

**Conclusion:**

Perception of unimpaired performance of ADL is a crucial barrier to appropriate help-seeking behavior after acute stroke, leading to undertreatment and less improvement in clinical symptoms. Thus, beyond improving the public’s knowledge of stroke symptoms, future public education campaigns should focus on the need for calling the EMS in case of stroke symptoms even if daily activities do not seem to be severely impaired.

## Introduction

Stroke is a leading cause of death and disability worldwide (1), and, apart from individual suffering, causes a pronounced economic burden for society (2). Intravenous thrombolysis and intra-arterial treatment are recommended as effective treatments for acute ischemic stroke (3). However, both of these recanalizing treatments are extremely time-sensitive: the magnitude of benefit diminishes rapidly as delay before treatment increases (“time is brain”) (4–6).

Treatment delays and associated undertreatment are mainly caused by problems that occur in the prehospital phase of acute stroke management, and patients’ inappropriate help-seeking behavior is the main such problem (7, 8). A large number of studies have clearly shown that calling the Emergency Medical Services (EMS) rather than visiting a general practitioner (GP) or using private transportation to the hospital reduces delays and increases treatment rates (9–15). Disappointingly, most public education campaigns that have been performed to improve patients’ help-seeking behavior achieved only small transient or no effects (16–20). One randomized study of the effects of community stroke education intervention on frequency of stroke treatment found positive effects, but mainly because of improved EMS and hospital staff performance, rather than patients’ behavior (21). This emphasizes the need for a better understanding of patients’ subjective perception and related response in case of stroke symptoms.

Studies investigating the factors affecting appropriate EMS use have found that elevated age, high severity of stroke symptoms, acute onset of symptoms, and the presence of a bystander are predictors of the decision to call the EMS (10, 13–15, 22–26). However, only a few studies have investigated the effects of psychological factors, which could be modified by educational campaigns (9). So far, correct recognition of stroke symptoms (24, 25) and perception of the urgency of the stroke event have been related positively to EMS use (10, 22, 26) and to shorter prehospital delays (27, 28).

However, the effect of concrete behavioral factors, such as patients’ subjective perception of impairment in activities of daily living (ADL), on EMS use has not been studied so far. Here, in search of new options for improving future public education campaigns, we investigated the effect of psychological factors on the decision to call the EMS by acute stroke patients. Moreover, we assessed the effects of these factors on prehospital delays, treatment rates, and indicators of clinical improvement.

## Patients and methods

### Study design

This prospective, cross-sectional study was conducted from August 2010 to December 2014. It consecutively enrolled patients who were admitted to the Department of Neurology, University of the Saarland, Homburg, Germany, and the Department of Neurology of the associated Teaching Hospital, Saarbrücken, Germany. Note that patients were only considered for participation if they were admitted during working hours of the psychologists. Thus, the 2,295 patients considered for participation were not the only ones who were admitted in this time period. No power analysis took place ahead of sampling. No contaminating public education campaigns targeting help-seeking behavior took place during or before this study.

**Figure 1:**
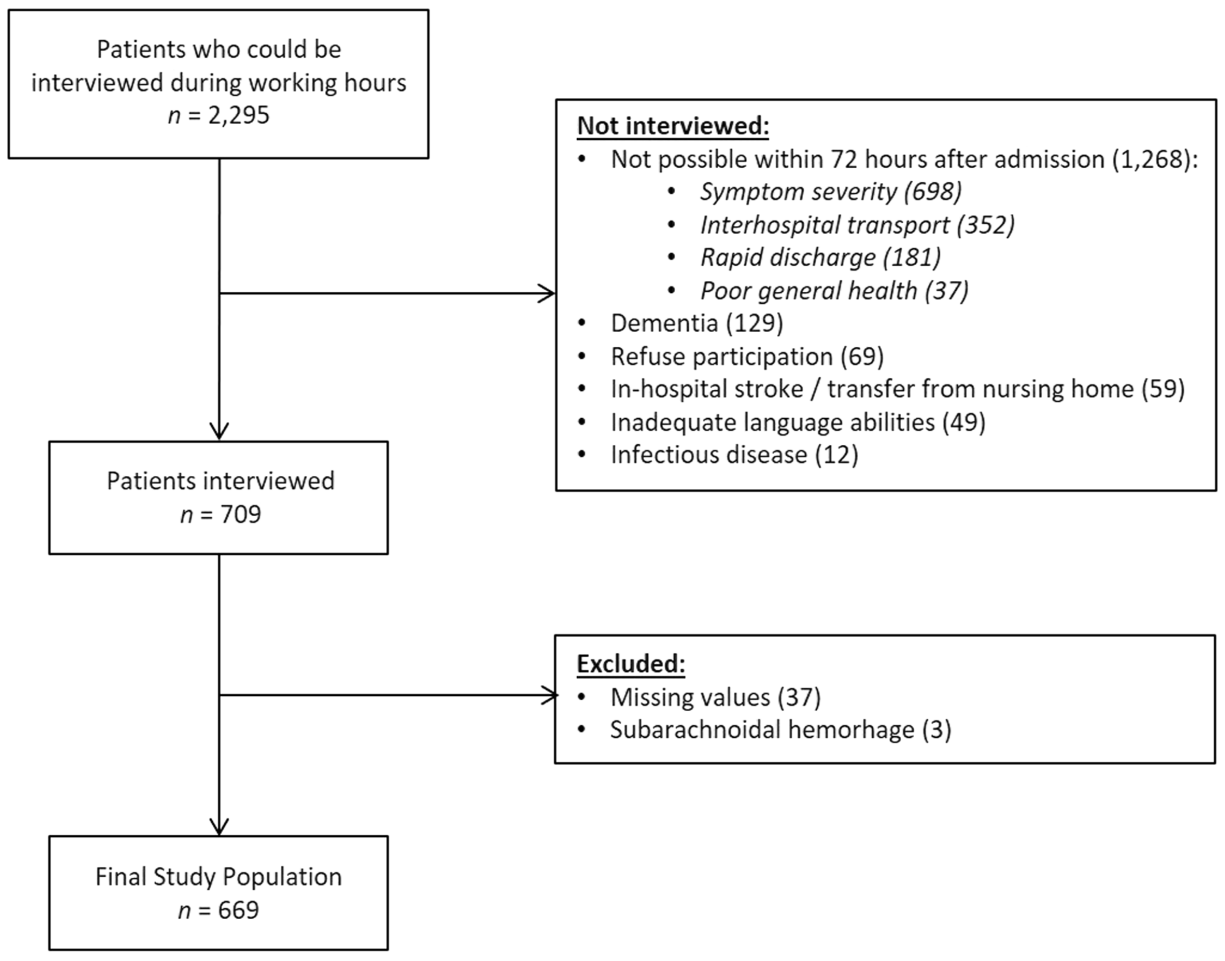
CONSORT, Consolidated standards of reporting trials.

Patients were included if they were at least 18 years old and had experienced an ischemic stroke, a transient ischemic attack, or an intracerebral hemorrhage. Patients with subarachnoid hemorrhage were interviewed but not considered in data analysis. Patients were also excluded in case of absence of informed consent, inability to be interviewed within 72 h after hospital admission, in-hospital stroke, secondary transfer from a first receiving hospital or from a nursing home, and the presence of dementia or inadequate language abilities (Consolidated Standards of Reporting Trials [CONSORT] diagram, Figure 1).

### Interview procedure and psychological assessment

To avoid the effect of losing memories of this event over time, all patients were interviewed within a 72-h window after hospital admission. The interviews lasted for 40 to 45 min and were conducted verbally, face-to-face, by trained psychological interviewers.

The structured interview collected demographic and medical data: time and location of the acute stroke, acute stroke symptoms, acuity and disease severity, personal or family history of stroke, symptom knowledge, lifestyle habits, previous major illnesses, and psychological factors. Psychological factors included a variety of cognitive appraisal factors of stroke symptoms: (1) subjective perception of impairment in the performance of ADL; (2) subjective perception of severity of stroke symptoms; (3) subjective experience of control of stroke symptoms; (4) and subjective recognition of symptoms as a stroke. Each factor was measured with a five-point Likert-type scale, ranging from 1 (very low) to 5 (very high).

Symptom knowledge was evaluated by the number of stroke symptoms that patients could spontaneously list, including paralysis/weakness, numbness, vision impairment, vertigo/dizziness, aphasia, amnesia, apraxia, headache, and disturbance of consciousness. Improvement of clinical symptoms in ischemic stroke patients was measured as the difference between modified Rankin scale (mRS) scores assessed at hospital admission and at hospital discharge. Higher values indicated more improvement.

### Ethical aspects

The study protocol, the informed consent document, and the subject information document were approved by the Ethics Committee of the Saarland Medical Association (AZ-Nr.: 101/10). All patients provided written informed consent for participation.

### Statistical analyses

Univariate and multivariate logistic and linear regression analyses were used to investigate the effects of the various psychological factors on the decision to call the EMS and on clinical improvement. As a measure of effect size, we calculated the actual change in the Hosmer and Lemeshow *R^2^* (*R_L_^2^*) for each predictor so that we could identify the most important cognitive appraisal factor (29). Each cognitive appraisal factor was dichotomized (1–3, low degree; 4–5, high degree) on the basis of patients’ ratings during the interview.

Furthermore, to investigate the indirect effect of cognitive appraisal on improvement of disease severity, mediated by EMS use, prehospital delay times, and thrombolytic treatment rates, we conducted a full serial mediation structural equation model using the “lavaan” package in R (30). Due to the focus in thrombolytic treatment, only patients with ischemic stroke were included in this analysis. Prehospital delay times (time from symptom onset to hospital admission) were logarithmically transformed. Parameter estimates and bootstrapped standard errors (5,000 draws) were calculated with a diagonally weighted least squares (DWLS) estimator. Direct and indirect effects were determined with bias-corrected and accelerated bootstrap confidence intervals (CI). To assess the absolute fit of the mediation model, we reported a range of model-fit indices: Root Mean Square Error of Approximation (RMSEA; values <0.05 indicate a good fit), comparative fit index (CFI; values >0.95); Tucker-Lewis index (TLI; values >0.95); and standardized root mean squared residual (SRMR; values ≤0.08). All tests were two-tailed with a significance level of α = 0.05 and were performed with RStudio 3.6.1 software (R Foundation for Statistical Computing, Vienna, Austria).

## Results

Of 2,295 acute stroke patients admitted during the study period, 709 were interviewed during working hours within 72 h after admission. Of these, 40 were excluded from data analysis because of missing data or subarachnoid hemorrhage (Figure 1). A detailed description of the final study population (*n* = 669), grouped by EMS use, is presented in Table 1.

**Table 1:** Patient characteristics by EMS use.^a^

	Total (*n* = 669)	EMS use (*n* = 326)	No EMS use (*n* = 343)
General			
Help-Seek Strategy, *n* (%)			
*Call EMS*	326 (48.7)	326 (100)	
*Call/Visit GP*	50 (7.5)		50 (14.6)
*Drive Self*	33 (4.9)		33 (9.6)
*Be Driven*	260 (38.9)		260 (75.8)
Age, median (range)	64 (19–89)	67 (19–89)	63 (20–87)
Women, *n* (%)	273 (40.8)	127 (39.0)	146 (42.6)
Education, *n* (%)			
*low*	367 (54.6)	194 (59.5)	173 (50.4)
*moderate*	183 (27.4)	86 (26.4)	97 (28.3)
*high*	119 (17.8)	46 (14.1)	73 (21.3)
Currently smoking, *n* (%)	159 (23.8)	77 (23.6)	82 (23.9)
BMI, median (range)	27.1 (15.9–46.4)	27.1 (15.9–46.3)	27.0 (18.3–46.4)
Stroke history, *n* (%)	150 (22.4)	75 (23.0)	75 (21.9)
Stroke family history, *n* (%)	275 (41.1)	125 (38.5)	150 (45.0)
Symptom knowledge, median (range)	2 (0–7)	2 (0–6)	2 (0–7)
Alone at onset, *n* (%)	160 (23.9)	77 (23.6)	83 (24.2)
Home at onset, *n* (%)	479 (71.6)	251 (77.0)	228 (66.5)
Medical			
Diagnosis, *n* (%)			
*Ischemic stroke*	410 (61.3)	202 (62.0)	208 (60.6)
*Hemorrhagic stroke*	12 (1.8)	5 (1.5)	7 (2.0)
*TIA*	247 (36.9)	119 (36.5)	128 (37.3)
NIHSS, median (range)	1 (0–40)	2 (0–40)	1 (0–9)
Sudden onset, *n* (%)	555 (83.0)	290 (89.0)	265 (77.3)
Persistent symptoms, *n* (%)	535 (80.0)	284 (87.1)	251 (73.2)
Psychological			
Perceived impairment, *n* (%)			
*low*	277 (41.4)	98 (30.1)	179 (52.2)
*high*	392 (58.6)	228 (69.1)	164 (47.8)
Perceived severity, *n* (%)			
*low*	384 (57.4)	171 (52.5)	213 (62.1)
*high*	285 (42.6)	155 (47.5)	130 (37.9)
Perceived control, *n* (%)			
*low*	543 (81.2)	274 (84.0)	269 (78.4)
*high*	126 (18.8)	52 (16.0)	74 (21.6)
Recognition, *n* (%)			
*low*	434 (64.9)	209 (64.1)	225 (65.6)
*high*	235 (35.1)	117 (35.9)	118 (34.4)
Treatment			
Prehospital delay (hours), median (range)	3.58 (0.22–425.12)	1.80 (0.32–144.67)	7.07 (0.22–425.12)
Thrombolysis, *n* (%)^b^	60 (14.6)	50 (24.8)	10 (4.8)

^a^Categorical variables are presented as absolute values with percentages; n (%). Continuous variables are presented as medians with ranges.

^b^Thrombolysis rates refer only to patients with ischemic stroke.

BMI, body mass index; EMS, Emergency Medical Services; GP, general practitioner; NIHSS, National Institutes of Health Stroke Scale; TIA, transient ischemic attack.

Among the 669 patients (273 [40.8%] women) in the study population, 410 (61.3%) had experienced an ischemic stroke; 247 (36.9%), a transitory ischemic attack; and 12 (1.8%), an intracerebral hemorrhage.

### Decision to call the EMS

Only 326 (48.7%) patients initially called the EMS. Among the remaining 343 patients, 50 (14.6%) contacted their GP, 33 (9.6%) drove to the hospital on their own, and 260 (75.8%) were driven to the hospital by another person.

The multivariate adjusted logistic regression analysis (Table 2) showed that, among the various cognitive appraisal factors studied, the only factor that independently predicted EMS use was subjective perception of impairment in performing ADL. Thus, patients who perceived a high impairment in performing ADL were 1.86-fold (odds ratio [OR], 1.86 [95% CI, 1.31–2.66]; *p* = 0.001) more likely to call the EMS than to use another help-seeking strategy, regardless of stroke severity, symptom knowledge, demographic, situational, medical, or other psychological factors.

**Table 2:** Univariate and multivariate binary logistic regression models; dependent variable: EMS use.

		Univariate	Multivariate		
	OR (95% CI)	*p* value	OR (95% CI)	*p* value	R_*L*_^*2*^_Change
General					
Age	1.02 (1.01–1.03)	<0.001	1.02 (1.01–1.03)	0.007	0.008
Woman	0.86 (0.63–1.17)	0.343	0.80 (0.57–1.13)	0.201	0.002
Education					
*low*	Ref.	Ref.	Ref.	Ref.	0.002
*moderate*	0.79 (0.55–1.13)	0.195	1.06 (0.71–1.58)	0.790	
*high*	0.56 (0.37–0.85)	0.007	0.75 (0.46–1.21)	0.239	
Previous stroke	1.07 (0.74–1.54)	0.724	1.02 (0.68–1.54)	0.911	<0.001
Symptom knowledge	0.90 (0.81–1.01)	0.066	0.99 (0.87–1.12)	0.838	<0.001
Alone at onset	0.97 (0.68–1.38)	0.861	1.02 (0.69–1.50)	0.938	<0.001
Home at onset	1.68 (1.20–2.38)	0.003	1.48 (1.02–2.16)	0.042	0.004
Medical					
NIHSS score	1.20 (1.13–1.28)	<0.001	1.16 (1.09–1.25)	<0.001	0.027
Sudden onset	2.37 (1.56–3.67)	<0.001	2.01 (1.28–3.22)	0.003	0.010
Persistent symptoms	2.48 (1.67–3.74)	<0.001	1.82 (1.18–2.83)	0.007	0.008
Psychological					
Perceived impairment	2.54 (1.85–3.50)	<0.001	1.86 (1.31–2.66)	0.001	0.013
Perceived severity	1.49 (1.09–2.02)	0.012	1.33 (0.91–1.96)	0.143	0.002
Perceived control	0.69 (0.46–1.02)	0.064	0.96 (0.61–1.51)	0.861	<0.001
Recognition of stroke	1.07 (0.78–1.47)	0.687	0.78 (0.53–1.15)	0.209	0.002

Dependent variable: help-seeking behavior (1, EMS use; 0, other strategy).

CI, confidence interval; OR, odds ratio; NIHSS, National Institutes of Health Stroke Scale; Ref., reference.

The actual change in *R_L_^2^* (*R_L_^2^_Change*) for each of the cognitive evaluation factors was calculated as the difference between the *R_L_^2^* of the total model (0.117) and the *R_L_^2^* of the model without the specific factor. The *R_L_^2^_Change* was highest for subjectively perceived impairment in performance of ADL (0.013) and lowest for subjectively perceived severity of symptoms (0.002), stroke recognition (0.002), and subjective perception of control of stroke symptoms (<0.001). Thus, the perception of impairment in performing ADL was the key psychological determinant of patients’ decision to call the EMS.

Among the non-psychological factors, those independently associated with EMS use were elevated age (OR, 1.02 [95% CI, 1.01–1.03]; *p* = 0.007); being at home (OR, 1.48 [95% CI, 1.02–2.16]; *p* = 0.042); high National Institutes of Health Stroke Scale (NIHSS) scores (OR, 1.16 [95% CI, 1.09–1.25]; *p* < 0.001); sudden onset of symptoms (OR, 2.01 [95% CI, 1.28–3.22]; *p* = 0.003); and persistence of symptoms (OR, 1.82 [95% CI, 1.18–2.83]; *p* = 0.007). Symptom knowledge did not have a significant impact on EMS use (OR, 0.99 [95% CI, 0.87–1.12]; *p* = 0.838).

In order to make our results more generalizable to major stroke populations, we repeated the exactly same multivariate adjusted logistic regression analysis, using only the subgroup of 235 patients (35%), who correctly identified their symptoms as stroke. This also revealed that patients who perceived high impairment in performing ADL were 2.94-fold more likely to use the EMS (OR, 2.94 [95% CI, 1.51–5.88]; *p* = 0.002). As in the main analysis, none of the other psychological factors reached significance. Note that NIHSS scores were significantly higher for those who correctly identified their symptoms as stroke than for those who did not, as indicated by two-sided independent t-test [*t*(667) = 1.99; *p* = 0.048].

As perceived impairment in ADL might belong to the older study population, we repeated the exactly same multivariate adjusted logistic regression analysis, stratified by median age (64 years). These analyses revealed that younger patients (age ≤ 64, n = 343) who perceived high impairment in performing ADL were 2.11-fold more likely to use the EMS (OR, 2.11 [95% CI, 1.27–3.54]; *p* = 0.004). Older patients (age > 64, n = 326) who perceived high impairment in performing ADL were 1.79-fold more likely to use the EMS (OR, 1.79 [95% CI, 1.07–3.01]; *p* = 0.028). Thus, our major result is not moderated by age.

### Improvement in clinical disease severity

Among ischemic patients (n = 410), analysis indicated that patients who subjectively experienced high impairment in ADL had a higher degree of improvement in mRS scores from hospital admission to discharge (*b* = 0.40, *t*(395) = 2.93, *p* = 0.004), independent of demographic, situational, medical, or other psychological factors (Table 3). None of the other predictors and covariates studied reached statistical significance.

**Table 3:** Univariate and multivariate OLS regression models; dependent variable: Modified Rankin Scale improvement.

	Univariate		Multivariate	
	*b* value	*p* value	*b* value	*p* value
General				
Age	−0.00	0.642	−0.00	0.602
Woman	−0.14	0.273	−0.17	0.207
Education				
*low*	Ref.	Ref.	Ref.	Ref.
*moderate*	−0.01	0.956	0.06	0.691
*high*	−0.15	0.380	−0.10	0.580
Previous stroke	−0.06	0.708	−0.02	0.915
Symptom knowledge	−0.03	0.497	−0.03	0.550
Alone at onset	0.05	0.730	0.10	0.476
Home at onset	0.08	0.566	0.06	0.663
Medical				
Sudden onset	0.20	0.221	0.07	0.684
Persistent symptoms	0.19	0.228	0.17	0.310
Psychological				
Perceived impairment	0.44	0.001	0.40	0.004
Perceived severity	0.11	0.364	0.07	0.615
Perceived control	−0.13	0.389	−0.03	0.853
Recognition of stroke	−0.09	0.495	−0.16	0.264

Dependent variable: improvement in modified Rankin scale score.

OLS, ordinary least squares; Ref., reference.

**Figure 2:**
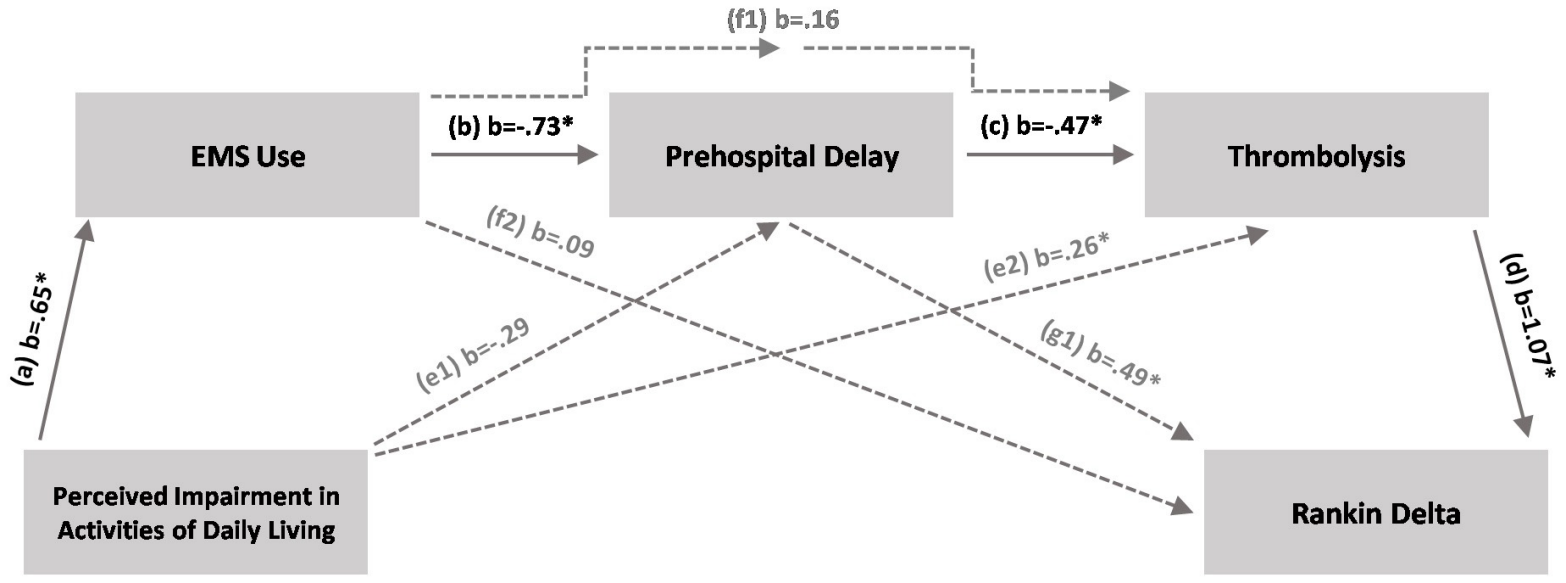
Full serial mediation model. The full serial mediation model shows that perceptions of impaired activities of daily living (ADL) lead to a higher likelihood of EMS use, which in turn decreases prehospital delay times and increases the likelihood of thrombolytic treatment leading to higher modified Rankin scale score improvement [indirect path 1, a × b × c × d (solid lines)]. *Indicates confidence interval different from zero. EMS, Emergency Medical Services.

We then tested whether the relationship between perceived impairment in performing ADL and improvement in mRS scores was serially mediated by EMS use, prehospital delays, and thrombolytic treatment rates (Figure 2). Fit statistics indicated an acceptable model fit (RMSEA = 0.000; *p* = 0.866; CFI = 1.000; TLI = 1.005; SRMR = 0.148).

Path analysis indicated a statistically significant direct path between perceived impairment in ADL and EMS use (*b* = 0.65 [95% CI, 0.38–0.90]). Perceived impairment in ADL explained 8.8% of the variance in EMS use. Furthermore, EMS use significantly predicted prehospital delays (*b*, −0.73 [95% CI, −0.88 to −0.57]); EMS use, together with perceived impairment in ADL (not statistically significant), explained 29.9% of the variance in delays. In turn, prehospital delays (*b*, −0.47 [95% CI, −0.58 to −0.34]) and perceived impairment in ADL (*b*, 0.26 [95% CI, 0.08–0.75]), together with EMS use (not statistically significant), significantly predicted the likelihood of thrombolytic treatment and explained 51.9% of the treatment variance. Finally, the difference in mRS scores was significantly predicted by thrombolytic treatment rates (*b*, 1.07 [95% CI, 0.25–1.72]) and prehospital delay times (*b*, 0.49 [95% CI, 0.09–0.91]); these factors, together with EMS use (not statistically significant), explained 76.1% of the variance in difference between mRS scores. Importantly, the indirect path 1 (a × b × c × d) significantly differed from zero (*b*, 0.24 [95% CI, 0.05–0.50]), a finding indicating a causal relationship between subjectively perceived impairment in performing ADL and mRS improvement, serially mediated by EMS use, prehospital delay times, and thrombolytic treatment rates.

## Discussion

Psychological factors determine patients’ appropriate help-seeking behavior as a precondition for fastest possible treatment onset and good outcome. This study showed that the perception of low impairment in performing ADL is a key psychological factor associated with the decision to not call the EMS, treatment delays, undertreatment, and, finally, poorer clinical improvement.

Consistent with earlier studies, this study found that roughly half of the acute stroke patients directly contacted the EMS (22, 23, 31). As in our study, it has been well documented that elevated age and more severe stroke is associated with EMS use (13, 15) potentially because older patients have a caregiver around and because they are not prone to drive to hospital on their own. Further, it has been discussed previously that patients with more severe strokes have more obvious symptoms and are therefore less uncertain about urgency to call the EMS (23). Moreover, our findings mirror the results of earlier studies pointing out that EMS use and earlier hospital admission are associated with sudden onset of symptoms that remain stable over time (24, 32).

However, this study shows that, among the variety of psychological factors investigated, only perception of impairment in performing ADL was associated with EMS use, independent of stroke severity, demographic, situational, and medical covariates. Subgroup analyses also showed that this association is not moderated by age, as it could be possible that survivors without impairment in ADL belong to the younger age group within the sample. Further, in order to make our results more generalizable toward stroke populations, we found that the association between perceived impairment in ADL and EMS use also stands for only those who correctly recognized their symptoms as stroke. Thus, patients use a behavioral performance criterion (i.e., ability to manage ADL) in deciding whether to call the EMS. Patients who do not perceive impairment in managing ADL use more autonomous but detrimentally inappropriate help-seeking strategies, such as driving to the hospital by car or contacting a GP, instead of calling the EMS. This result also fits within a model of patients’ decision-making process in stroke, proposed by Moloczij and colleagues 2008 (33). Therefore, besides “making sense of symptoms” one major aspect of patients’ decision-making is the ability to “maintain a sense of normality” (i.e., mental/physical self-management, maintain responsibilities and commitments, maintain independence). It seems natural that the perceived inability to manage ADL stands in contrast to a maintained sense of normality.

The clinical importance of perception of low impairment in performing ADL is further supported by the results of the mediation analysis, which showed that for ischemic stroke patients this psychological factor strongly decreases the likelihood of EMS use, thereby increasing prehospital delays that lead to undertreatment and, finally, to poorer outcome.

So far, public education campaigns have unfortunately achieved only limited transient or no effects on patients’ help-seeking behavior (16–20), potentially because they focus mainly on knowledge of stroke symptoms rather than on subjective psychological factors (33, 34). The current study showed that perceived low impairment in performing ADL is a modifiable risk factor for a decision against calling the EMS that could be a novel target for future public education campaigns.

As a potential limitation, we did not interview potential bystanders about their subjective perceptions, although these persons may play an important role in help-seeking behavior (25, 26, 35). Nevertheless, our study showed that the presence of bystanders did not affect patients’ help-seeking behavior. Moreover, we included only patients who could be interviewed within 72 h after admission, a criterion that may have excluded patients with the most severe symptoms, which is also reflected in the relatively low NIHSS scores in the study population. Thus, the present study may not be representative for patients who have higher NIHSS scores. However, subgroup analysis including only patients, who identified their symptoms as stroke, and had higher NIHSS scores than those who did not, revealed that perceived impairment in ADL is still the only psychological factor that independently predicts EMS use. This may make the results more generalizable toward stroke populations. Further, this strict time criterion was considered to be important to accurately capture patients’ memories of the acute event, as we wanted to capture the very first appraisal in the acute situation which might be crucial for decision-making. Another limitation is the relatively long interval from data collection up to now. However, in contrast to improvement in delays in in-hospital stroke management, delays due to patients’ inappropriate help-seeking behavior did not significantly change in the past years (36). Further, no educational campaigns took place in between data acquisition and writing of this paper.

In conclusion, perceived impairment in the performance of ADL may play a key role in help-seeking behavior, with marked effects on treatment delays, treatment rates, and clinical improvement. Future public education campaigns could, therefore, be more successful if they target the risk of relying on the misleading perception of unaffected performance of ADL, e.g., by communicating that “Stroke is life-threatening, even if you do not feel disabled. Therefore, always call the EMS!.” Future research should clarify if considering perceived impairment in ADL can boost the effect of public education.

## Data availability statement

The raw data supporting the conclusions of this article will be made available by the authors, without undue reservation.

## Ethics statement

The studies involving humans were approved by Ethics Committee of the Saarland Medical Association (Ethikkommission bei der Ärztekammer des Saarlandes; AZ-Nr: 101/10). The studies were conducted in accordance with the local legislation and institutional requirements. The participants provided their written informed consent to participate in this study.

## Author contributions

KF: conceptualization. LT, FM, and KF: design and methodology. MLu, AR, MG, and JW: data collection. LT: statistical analysis. KF: supervision, project administration. LT, MLe, and KF: writing the manuscript. All authors contributed to the article and approved the submitted version.

## Figures and Tables

**Table:** 

ADL	activities of daily living
BMI	body mass index
CFI	comparative fit index
CI	confidence interval
CONSORT	Consolidated Standards of Reporting Trials
DWLS	diagonally weighted least squares
EMS	Emergency Medical Services
GP	general practitioner
mRS	modified Rankin scale
NIHSS	National Institutes of Health Stroke Scale
OLS	ordinary least squares
OR	odds ratio
RMSEA	Root Mean Square Error of Approximation
SRMR	standardized root mean squared residual
TIA	transient ischemic attack
TLI	Tucker-Lewis index
